# Evaluation of Conventional Cardiovascular Risk Factors and Ordinal Coronary Artery Calcium Scoring in a Lung Cancer Screening Cohort

**DOI:** 10.3390/jcdd11010016

**Published:** 2024-01-05

**Authors:** Piotr Kasprzyk, Aleksandra Undrunas, Katarzyna Dziadziuszko, Robert Dziedzic, Krzysztof Kuziemski, Edyta Szurowska, Witold Rzyman, Tomasz Zdrojewski

**Affiliations:** 1 First Department of Cardiology, Medical University of Gdańsk, 80-210 Gdańsk, Poland; 2 Department of Preventive Medicine and Education, Medical University of Gdańsk, 80-210 Gdańsk, Poland; a.undrunas@gumed.edu.pl (A.U.); tz@gumed.edu.pl (T.Z.); 3 II Department of Radiology, Medical University of Gdańsk, 80-210 Gdańsk, Poland; katarzyna.dziadziuszko@gumed.edu.pl (K.D.); edyta.szurowska@gumed.edu.pl (E.S.); 4 Department of Thoracic Surgery, Medical University of Gdańsk, 80-210 Gdańsk, Polandwitold.rzyman@gumed.edu.pl (W.R.); 5 Department of Allergology and Pneumonology, Medical University of Gdańsk, 80-210 Gdańsk, Poland; krzysztof.kuziemski@gumed.edu.pl

**Keywords:** lung cancer screening, cardiovascular risk, coronary artery calcium score

## Abstract

(1) Background: Lung cancer screening (LCS) consists of low-dose computed tomography (LDCT) results to reduce lung cancer-related mortality. The LCS program has a unique opportunity to impact CVD mortality by providing tools for CVD risk assessment and implementing preventative strategies. In this study, we estimated standardized CVD risk (SCORE) and assessed the prevalence of coronary artery calcium (CAC) in a Polish LCS cohort. (2) Methods: In this observational study, 494 LCS participants aged 50–79 years with a cigarette smoking history of at least 30 pack-years were included. Medical history, anthropometric measurements, blood pressure measurements, serum glucose, and cholesterol levels were assessed in one visit. CVD risk assessment using SCORE tables was performed. The results were compared to the general population (NATPOL 2011 study). On LDCT scans, CAC was classified using an Ordinal Score ranging from 0 to 12. (3) Results: The prevalence of classic cardiovascular risk factors was very high. Among study participants, 83.7% of men and 40.7% of women were classified with a very high CVD SCORE risk (>10%). CAC was reported in 190 (47%) participants. Calcification was categorized as severe (CAC ≥ 4) in 84 (21%) participants. (4) Conclusions: Due to the high cardiovascular risk, intensive preventive strategies are recommended for LCS participants.

## 1. Introduction

Tobacco dependence is a significant social and health problem caused by the pharmacological effects of nicotine [1]. Smoking is known to be a severe risk factor for many heterogeneous diseases. Smoking is the leading cause of lung cancer and chronic obstructive pulmonary disease (COPD). It also significantly increases the risk of cardiovascular disease (CVD). According to a WHO report [2], these three diseases are the leading causes of noncommunicable diseases and deaths worldwide [3]. However, they are preventable. In the USA [4], (National Lung Screening Trial, NLST) and Europe [5,6,7] (NELSON, UKLS, and MILD studies), effective attempts have been made to significantly reduce all-cause mortality and lung cancer-related mortality in current and former smokers using low-dose computed tomography (LDCT) screening. In 2013, in response to an NLST study, the US Preventive Services Task Force recommended lung cancer screening (LCS) using low-dose computed tomography [8]. Several years later, similar recommendations have also been developed in Europe [9,10]. Despite the obvious benefits, implementing lung cancer screening is a complex organizational and economical task. It may pose a significant financial burden on healthcare systems. Furthermore, patients who qualify for lung cancer screening programs may also be characterized by a high prevalence of other smoking-related diseases [11]. High exposure to tobacco in the LCS population can significantly increase the risk of cardiovascular comorbidities (acute coronary syndrome, stroke). LCS presents a unique opportunity to evaluate cardiovascular status using classic cardiovascular risk factors assessment (SCORE) and additional information from chest LDCT images, such as Coronary Artery Calcification (CAC). CAC has been shown to predict cardiovascular events and death [12,13] and can be used as another accurate indicator for identifying patients at high cardiovascular risk. In this group, conducting lung cancer screening and preventing cardiovascular diseases simultaneously appears justified [14]. Implementing preventive actions may also enhance the health significance and cost-effectiveness of LCS. However, cardiovascular risk varies significantly between different populations and countries. There is a lack of studies in the LCS population for precisely determining cardiovascular risk. In this study, we assessed classic cardiovascular risk factors and coronary artery calcifications using LDCT in a Polish cohort participating in lung cancer screening.

## 2. Materials and Methods

The population analysis was based on results obtained from a lung cancer screening trial MOLTEST-BIS cohort conducted at the Medical University of Gdańsk. They were compared to the general population sample from the NATPOL 2011 study. A MOLTEST-BIS program was designed to assess lung cancer screening effectiveness using low-dose computed tomography [15]. A total of 5534 healthy volunteers were enrolled in the program between January 2016 and December 2017. Based on the Lung Cancer Screening NCCN Clinical Practice Guidelines [16], inclusion criteria included an age of 50–79 years with a smoking history of >30 pack-years. As part of the trial, 494 responders were examined for cardiovascular risk factors. During the visit, an interview was conducted using a standardized questionnaire. The survey contained detailed questions about medical history, including diagnosed diseases, smoking status, symptoms, medications, socio-economic status, physical activity, health-promoting behaviors, and the presence of risk factors for heart and vascular diseases. Afterward, anthropometric measurements (body weight, height, and waist, hip, and neck circumference) were obtained, and physical examinations, blood pressure measurements, and laboratory blood tests were conducted. Body mass index (BMI) and waist–hip ratio (WHR) were calculated from anthropometric measurements. Blood pressure measurements were performed according to the recommendations of the European Society of Cardiology. Measurements were obtained with an automatic sphygmomanometer (A&D Medical, model UA-787+, 4622 Runway Boulevard, Ann Arbor, MI 48108, USA). The mean of the second and third measurements was taken as a blood pressure value [17]. Hypertension was defined as systolic blood pressure ≥140 and/or diastolic blood pressure ≥90 or a history of antihypertensive therapy. During laboratory testing, fasting serum glucose, total cholesterol, LDL, HDL, and triglyceride levels were measured. Diabetes was diagnosed based on medical history (previously diagnosed with diabetes or antihyperglycemic therapy) or fasting serum glucose (≥126 mg/dL). Hyperlipidemia was diagnosed based on medical history (previously diagnosed with hyperlipidemia or lipid-lowering therapy) and LDL level ≥115 mg/dL. Based on the obtained results, the 10-year SCORE risk of death from cardiovascular diseases was calculated. According to the ESC Guidelines for Prevention of Cardiovascular Diseases, patients with a history of acute coronary syndrome, coronary revascularization, stroke, aortic aneurysm, peripheral arterial disease, type 2 diabetes, and chronic kidney disease were classified as being very high risk. The SCORE risk was calculated using the Pol-SCORE algorithm validated for the Polish population [18]. Participants were classified into low/moderate risk (SCORE <5%), high risk (SCORE <10%), and very high risk (SCORE ≥10%) groups. A representative sample of the Polish population was selected as the control group from the NATPOL 2011 cross-sectional and observational. In this study, we assessed the prevalence and control of CVD risk factors in Poland based on a representative sample of adults aged 18–79. The survey was conducted between January and August 2011. The study comprised a questionnaire, blood pressure (BP), and anthropometric measurements as well as a blood and urine sample collection. A more detailed methodological description, sample selection procedure, and examination methods were used to assess existing risk factors. The procedure for blood samples obtained in the NATPOL 2011 survey was published elsewhere [18]. The NATPOL study sample was matched in terms of age and sex to avoid statistical differences between the populations.

**Figure 1:**
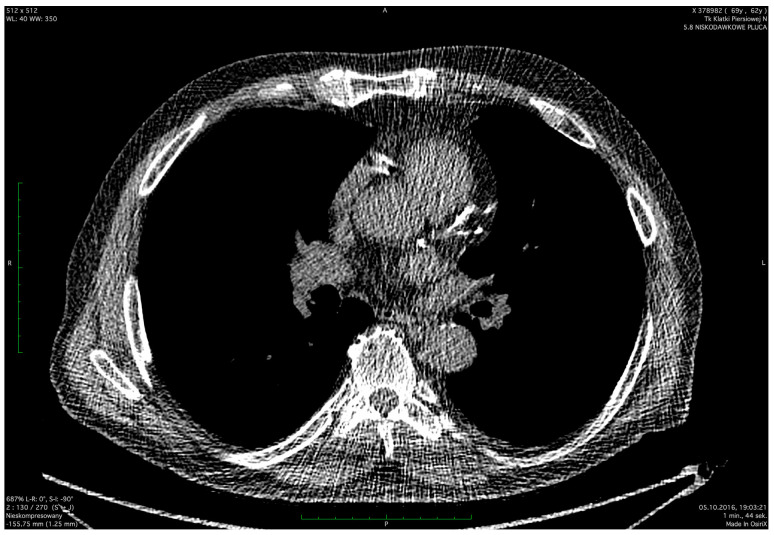
Massive calcifications in LM, LAD, and RCA. LM—left main, LAD—left anterior descending, RCA—right coronary artery.

**Figure 2:**
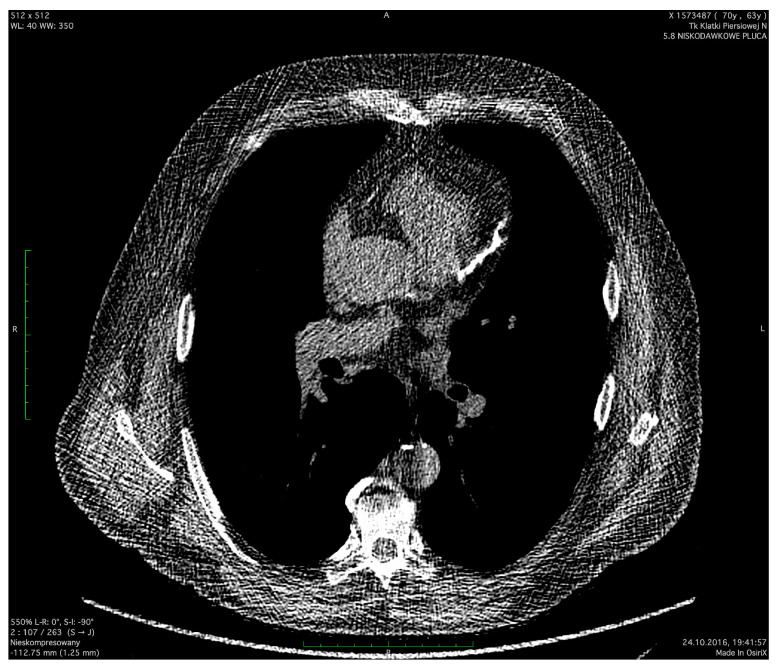
Long calcifications in LAD. LAD—left anterior descending.

The severity of coronary artery calcification was assessed by non-ECG-gated low-dose computed tomography (Figure 1 and Figure 2). LDCT scans were performed in the Radiology Department at the Medical University of Gdańsk using a 64-slice CT scanner (Lightspeed VCT, GE Healthcare, Milwaukee, WI, USA) without intravenous contrast agent administration. The scanning parameters were as follows: 120 kV tube voltage, 20–30 mAs tube current, and 1.25 mm slice thickness at the mediastinal window. CTs were not electrocardiographic (ECG) gated. The obtained CT images were not optimized for standard Agatston CAC scoring. In this context, the visual method for CAC assessment (ordinal scoring [19,20,21]) was chosen as an alternative approach. In this method, each coronary artery (left main coronary artery, left anterior descending artery, left circumflex artery, and right coronary artery) was scored from 0 to 3 depending on calcification length. In the absence of calcifications, a score of 0 was assigned. Calcifications covering less than a third of the vessel length were scored at 1 point. For calcifications involving one to two-thirds of the vessel, 2 points. In the case of calcifications over two-thirds of the vessel length, the score was 3 points. The total CAC score was the sum of the calcification scores in each artery, ranging from 0 to 12 points. For the purpose of statistical analyses, the study population was divided into the following CAC score subgroups: 0, 1–3, 4–6, and 7–12. This study was approved by the Independent Bioethics Committee for Scientific Research (no. NKBBN/173/2016).

### Statistical Analisys

Statistical calculations were performed in the R environment (version 3.2.3). Quantitative variables were presented using the median (25th–75th percentile). Qualitative variables were presented as numbers (n) and percentages (%). The assumption of a normal distribution was evaluated using the Shapiro–Wilk test. The assumption of homogeneity of variances was assessed using Leven’s test. A nonparametric Kruskal–Wallis test was used to compare groups. Post-hoc tests were performed using Dunn’s test with Bonferroni *p*-value correction. Differences between qualitative variables were tested using Fisher’s exact test. Values of *p* < 0.05 were considered significant.

## 3. Results

The general population characteristics and their anthropometric measurements are presented in Table 1 and Table 2. The average age was 63.5 years for men and 63 years for women. Significant differences in the education level between the study and control groups were found. Participants of the MOLTEST-BIS study had higher tertiary (31.6% vs. 20.8% in women and 28.9% vs. 15.4% in men) and secondary (41.5% vs. 27.2% in women and 48.1% vs. 42.3% in men) education compared to the general population. Moltest study participants smoked significantly more pack-years and had a higher number of active smokers than the general population.

Women in the Moltest study had a significantly lower body weight and BMI than women in the NATPOL study (body weight, 71.5 vs. 74; BMI, 27.5 vs. 28.8) and were characterized by a higher incidence of abdominal obesity (WHR ≥ 0.85 in 69% vs. 57%). Compared to women, reverse relationships were observed in the male population. Male smokers were characterized by a higher body weight (88.8 kg vs. 84.5 kg), BMI (29.1 vs. 28.2), and prevalence of abdominal obesity (WHR ≥ 1 in 45% vs. 29.5%). 

In Table 3, the prevalence of classic risk factors for coronary artery disease, such as type 2 diabetes, hypertension, and hypercholesterolemia is compared between the Moltest and NATPOL 2011 participants. There were no significant differences between established and newly diagnosed type 2 diabetes in the analyzed populations.

The overall prevalence of hypercholesterolemia was high but there were no significant differences between the groups. The proportion of men with previously diagnosed hypercholesterolemia was significantly higher in the Moltest study (62.4% vs. 33.8%), which resulted in a lower frequency of diagnosed de novo hypercholesterolemia (22.1% vs. 43.8%). There were no significant differences in total cholesterol, LDL, HDL, and triglyceride levels.

The prevalence and distribution of hypercholesterolemia in the female population showed a similar relationship to the male population.

The studied populations did not differ in terms of prevalence and mean values of blood pressure in men.

Furthermore, in the case of women, mean blood pressure values did not differ between populations but the overall prevalence and medical history of hypertension was statistically lower in the Moltest population.

The number of people classified as being at high or very high risk of cardiovascular (CV) death was considerable in the Moltest study (Table 4). Among women in the MOLTEST-BIS study, 20.9% were classified as having a very high (>10%) risk of CV death vs. 4.7% in the NATPOL study. Additionally, 27.3% of women in the Moltest study were classified as high risk (5–10%) vs. 15% in the NATPOL study. As many as 43.5% of men in the MOLTEST-BIS study were classified as having a very high risk of cardiovascular death vs. 15.5% in the NATPOL study. Conversely, the high-risk group included 14.6% of men in the Moltest study vs. 24.4% in the NATPOL study. In total, as many as 68% of women and 98.3% of the men assessed in the Moltest study were classified as high or very high risk, with a strong CVD factor.

Low-dose computed tomography showed coronary calcifications in as many as 47% of patients (Table 5). Moderate calcifications were found in 26% of the subjects, and severe calcifications (CAC ≥ 4) were found in 21% of participants.

## 4. Discussion

The Moltest study was designed to analyze the outcomes of CVD risk assessment in the LCS population. The resulting observations of the Polish cohort participating in lung cancer screening with low-dose computed tomography indicate that this population does not differ significantly from the general population in the prevalence of classic cardiovascular risk factors. Despite fewer differences in the assessed burden of risk factors, 68% of women and 98% of men were classified as having a high or very high risk of fatal cardiovascular complications according to the SCORE algorithm. Higher estimated cardiovascular risks for the study group than the control group were due to the first group’s significantly different smoking history. In addition, as many as 47% of the study participants had calcifications in the coronary arteries, and 21% of participants had calcifications categorized as severe, which corresponds with other studies [22,23,24]. The above data lead to considerations about implementing parallel preventive measures for cardiovascular diseases to reduce mortality. Furthermore, the high CVC prevalence in this group suggests the possibility of using CAC to personalize cardiovascular risk more precisely. Data on comorbidity in LCS cohorts vary by country and population screened [25]. However, according to our results, they indicate a high cardiovascular risk [26,27] and increased mortality due to cardiovascular diseases [28]. In the 10-year follow-up of participants in a Multi-Ethnic Study of Atherosclerosis (MESA), patients who met the inclusion criteria for LCS had more than a threefold higher risk of cardiovascular events (20.8%) [28] than the rest of the MESA cohort [29]. The above data suggest potential benefits associated with implementing CVD prevention, considering that prevention is most effective and cost-effective for those at high risk [30]. 

### 4.1. Cardiovascular Risk Estimation and Preventive Strategies

The lack of significant differences in the prevalence of hypertension, diabetes, and hypercholesterolemia in the study population compared to the control group from the general Polish population was surprising. In the Moltest group, the percentage of people with a history of cardiovascular disease risk factors (e.g., hypercholesterolemia) was even higher than in the general population. This is most likely due to the recruitment of study participants from a large urban center; therefore, they have easier access to basic medical care and are characterized by higher education and greater awareness than the general population. These findings are true for most screening populations [31]. However, in the Polish population, this effect is particularly strong [32]. In both groups, however, the absolute prevalence of these risk factors was high, which translates directly into a high SCORE risk. Despite the lack of significant differences in the prevalence and control of classic CVD risk factors, the Moltest population was characterized by a significantly higher 10-year risk of death due to cardiovascular diseases. A positive history of smoking is mainly associated with a higher risk of cardiovascular complications, so preventing smoking should be the main goal. Smoking cessation is a modifiable health behavior that can significantly impact mortality. This behavior is supported by pharmacological–behavioral anti-smoking interventions conducted by medical professionals. Furthermore, specific legislation implementing comprehensive tobacco control programs using price and non-price interventions can substantially raise smoking cessation rates and play a crucial role in anti-nicotine politics [33,34]. The importance of smoking cessation is particularly evident in the male population, of which almost 85% are in the very high-risk group. This finding nearly qualifies the entire study population for intensive preventive measures. The proportion of participants at very high cardiovascular risk may appear higher than expected compared to the results of other LCS cohorts. However, these studies were conducted on populations with a lower baseline risk than the Polish population and recruited younger participants [35,36]. On the other hand, the study results conducted on all-comer populations showing a higher degree of social deprivation were characterized by a very high CVD risk similar to the Polish population [26,27,36]. In addition, risk estimation using the SCORE calculator in such a high-risk group may be imprecise by overestimating or underestimating clinical risk in individual patients. Moreover, as new preventive treatments become available, this group’s uniformly high CVD risk may justify the need for more thorough risk stratification strategies.

### 4.2. Coronary Artery Calcification

Assessing calcifications in the coronary arteries from computed tomography can be very useful. There is evidence for CAC as a standalone risk factor [37,38], and when combined with classical CVD risk assessment [12,39], it can offer a much more refined and precise approach to risk stratification. An additional advantage of CAC assessment is that information on coronary artery calcification is obtained simultaneously during LDCT examination without involving additional financial and logistic resources. Shemesh et al., analyzing a group of 8782 people, showed that high CAC values correlate with the risk of cardiovascular death [20]. The subgroup with CAC ≥4 had an almost five times higher odds ratio for cardiovascular death than the group with CAC=0. It should be noted that the value of CAC ≥4 was found in 21% of the Polish cohort. The extent of coronary artery calcifications is important for patients with low and moderate risk, according to the classical SCORE algorithm, in whom a seemingly good prognosis can be expected. In the case of the Polish cohort, this problem mainly concerned women (32.1% had moderate SCORE risk). LDCT showing severe calcifications in moderate-risk patients allows reclassification to the high-risk group to be considered [27,40]. The added value of CAC was demonstrated by meta-analysis results [32], which showed that CAC detection improved patients’ adherence to medication and motivated them to change their health-related behavior. However, using CAC has some limitations. Firstly, due to image quality, the non-ECG-gated LDCT used in LCS does not allow calcification assessment with well-validated objective methods such as the Agatston method. In most screening studies, CAC was assessed by a subjective visual method. The visual assessment method has shown high agreement with the Agatston method results [19,21,41]. Despite several studies, the visual assessment method for calcifications requires further research to verify its clinical usefulness compared to classic cardiovascular risk assessment scales.

## 5. Study Limitations

Our study has several limitations. Firstly, our study was not randomized and was based on voluntary participation. Secondly, we did not include the entire cohort of LCS participants due to time constraints. Moreover, due to easier access to the LCS program, the study population mostly constituted people from a large urban agglomeration, which might have influenced the obtained results.

## 6. Conclusions

In conclusion, people participating in LCS are characterized by a high prevalence of CVD risk factors, a very high cardiovascular risk, and significant calcification of the coronary arteries, indicating that intensive interventions are required to prevent cardiovascular disease. The high risk of cardiovascular complications is mainly associated with a positive history of smoking, which should be prevented. Coronary artery calcification assessed by the visual method can be an additional indicator of estimating cardiovascular risk. Lung cancer screening programs address individuals with high cardiovascular risk and offer unique opportunities for parallel CVD prevention.

## Figures and Tables

**Table 1:** General characteristics.

	Women			Men		
	Moltest (N = 223)	NATPOL (n = 274)	*p*-Value	Moltest (N = 271)	NATPOL (n = 279)	*p*-Value
Age			0.303			0.265
Mean (SD)	63.0 (5.9)	62.5 (6.5)		63.5 (6.6)	63.0 (7.0)	
95% CI for mean	(62.4–63.6)	(61.7–63.3)		(62.9–64.1)	(62.1–63.8)	
Education			<0.001			<0.001
Primary	20.3%	36.9%		29.6%	57.3%	
Secondary	48.1%	42.3%		41.5%	27.2%	
Tertiary	31.6%	20.8%		28.9%	15.4%	
Smoking status			<0.001			<0.001
Current smoker	68.4%	22.3%		63.2%	29.4%	
Ex-smoker	31.6%	33.2%		36.8%	43.7%	
Never-smoker	0%	44.5%		0%	26.9%	
Pack-years			<0.001			<0.001
Mean (SD)	38.5	15.9		47.2 (18.0)	23.7 (17.3)	
95% Cl	(37.4–39.6)	(13.3–18.4)		(45.6–48.8)	(21.2–26.1)	

**Table 2:** Anthropometric measurements.

	Women			Men		
	Moltest (N = 223)	NATPOL (N = 274)	*p*-Value	Moltest (N = 271)	NATPOL (N = 279)	*p*-Value
Weight			0.022			<0.001
Mean (SD)	71.5 (13.3)	74.0 (14.5)		88.8 (15.3)	84.5 (14.3)	
95% CI for mean	(70.2–72.8)	(72.2–75.7)		(87.4–90.2)	(82.8–86.2)	
Waist circumference			0.432			0.031
Mean (SD)	93.2 (13.7)	94.1 (13.4)		104.5 (12.3)	102.6 (11.0)	
95% CI for mean	(91.9–94.6)	(92.5–95.7)		(103.4–105.7)	(101.3–103.9)	
BMI			<0.001			0.010
Mean (SD)	27.5 (4.8)	28.8 (5.3)		29.1 (4.8)	28.2 (4.3)	
95% CI for mean	(27–27.9)	(28.1–29.4)		(28.7–29.6)	(27.7–28.7)	
<18.5	2 (1%)	4 (1.5%)		1 (0.4%)	4 (1.5%)	
18.5–25	71 (32%)	65 (24%)		48 (17.6%)	47 (17%)	
25–30	85 (38%)	106 (39%)		119 (44%)	127 (46.5%)	
≥30	65 (29%)	97 (35.5%)		103 (38%)	96 (35%)	
WHR			0.002			<0.001
≥1 in men, ≥0.85 in women	153 (69%)	156 (57%)		123 (45%)	82 (29.5%)	
Waist circumference			0.840			0.463
≥94 in men, ≥80 in women	186 (83%)	226 (83%)		225 (83%)	226 (81%)	

BMI—Body mass index, WHR—waist hip ratio.

**Table 3:** Prevalence of diabetes, hypertension, and hypercholesterolemia.

	Women			Men		
	Moltest	NATPOL	*p*	Moltest	NATPOL	*p*
Diabetes, N	212	145		264	165	
History of diabetes, n (n/N%)	21 (9.9%)	18 (12.8%)	0.589	14.3%	12.9%	0.811
Newly diagnosed diabetes, n (n/N%)	5 (2.3%)	1 (0.7%)	0.230	2.7%	3.0%	0.852
Overall prevalence n (n/N%)	26 (12.2%)	19 (12.4%)	0.953	17.0%	16.3%	0.833
Hypercholesterolemia, N	223	144		271	160	
History of hypercholesterolemia, n (n/N%)	149 (66.8%)	73 (50.7%)	0.002	169 (62.4%)	54 (33.8%)	<0.001
Newly diagnosed hypercholesterolemia, n (n/N%)	44 (19.7%)	54 (37.5%)	<0.001	60 (22.1%)	70 (43.8%)	<0.001
Overall prevalence, n (n/N%)	193 (86.5%)	127 (88.2%)	0.645	229 (84.5%)	124 (77.5%)	0.068
Hypertension, N	223	274		271	279	
History of hypertension, n (n/N%)	40%	55%	0.0001	50%	48%	0.68
Newly diagnosed hypertension, n (n/N%)	21%	16%	0.206	21%	24%	0.46
Overall prevalence, n (n/N%)	61%	71%	0.003	71%	72%	0.88

**Table 4:** SCORE risk groups.

	Women		Men	
SCORE	Moltest (N = 187)	NATPOL (N = 386)	Moltest (N = 239)	NATPOL (N = 439)
<5%	60 (32.1%)	187 (48.4%)	4 (1.7%)	100 (22.8%)
5–10%	51 (27.3%)	58 (15.0%)	35 (14.6%)	107 (24.4%)
>10%	39 (20.9%)	18 (4.7%)	104 (43.5%)	68 (15.5%)
CVD	37 (19.8%)	123 (31.9%)	96 (40.2%)	164 (37.4%)

SCORE—Systematic Coronary Risk Evaluation, CVD—previous cardiovascular disease.

**Table 5:** CAC score distribution in lung cancer screening participants.

	CAC			
	0	1–3	4–6	7–12
Number of observations (n)	214 (53%)	106 (26%)	48 (12%)	36 (9%)

CAC—coronary artery calcium.

## Data Availability

Data available upon request due to restrictions, privacy, and ethical restrictions.

## References

[1] Alpert H.R., Agaku I.T., Connolly G.N. (2016). A Study of Pyrazines in Cigarettes and How Additives Might Be Used to Enhance Tobacco Addiction. Tob. Control.

[2] WHO (2010). Global Status Report on Noncommunicable Diseases 2010.

[3] (2015). Global, Regional, and National Age–Sex Specific All-Cause and Cause-Specific Mortality for 240 Causes of Death, 1990–2013: A Systematic Analysis for the Global Burden of Disease Study 2013. Lancet.

[4] The National Lung Screening Trial Research Team (2013). Results of Initial Low-Dose Computed Tomographic Screening for Lung Cancer. N. Engl. J. Med..

[5] de Koning H.J., van der Aalst C.M., de Jong P.A., Scholten E.T., Nackaerts K., Heuvelmans M.A., Lammers J.-W.J., Weenink C., Yousaf-Khan U., Horeweg N. (2020). Reduced Lung-Cancer Mortality with Volume CT Screening in a Randomized Trial. N. Engl. J. Med..

[6] van Iersel C.A., de Koning H.J., Draisma G., Mali W.P.T.M., Scholten E.T., Nackaerts K., Prokop M., Habbema J.D.F., Oudkerk M., van Klaveren R.J. (2007). Risk-Based Selection from the General Population in a Screening Trial: Selection Criteria, Recruitment and Power for the Dutch-Belgian Randomised Lung Cancer Multi-Slice CT Screening Trial (NELSON). Int. J. Cancer.

[7] Pastorino U., Silva M., Sestini S., Sabia F., Boeri M., Cantarutti A., Sverzellati N., Sozzi G., Corrao G., Marchianò A. (2019). Prolonged Lung Cancer Screening Reduced 10-Year Mortality in the MILD Trial: New Confirmation of Lung Cancer Screening Efficacy. Ann. Oncol. Off. J. Eur. Soc. Med. Oncol..

[8] Moyer V.A., on behalf of the U.S. (2014). Preventive Services Task Force. Screening for Lung Cancer: U.S. Preventive Services Task Force Recommendation Statement. Ann. Intern. Med..

[9] Kauczor H.-U., Baird A.-M., Blum T.G., Bonomo L., Bostantzoglou C., Burghuber O., Čepická B., Comanescu A., Couraud S., Devaraj A. (2020). ESR/ERS Statement Paper on Lung Cancer Screening. Eur. Radiol..

[10] Oudkerk M., Devaraj A., Vliegenthart R., Henzler T., Prosch H., Heussel C.P., Bastarrika G., Sverzellati N., Mascalchi M., Delorme S. (2017). European Position Statement on Lung Cancer Screening. Lancet Oncol..

[11] Team N.L.S.T.R., Aberle D.R., Adams A.M., Berg C.D., Clapp J.D., Clingan K.L., Gareen I.F., Lynch D.A., Marcus P.M., Pinsky P.F. (2010). Baseline Characteristics of Participants in the Randomized National Lung Screening Trial. J. Natl. Cancer Inst..

[12] Greenland P., LaBree L., Azen S.P., Doherty T.M., Detrano R.C. (2004). Coronary Artery Calcium Score Combined with Framingham Score for Risk Prediction in Asymptomatic Individuals. JAMA.

[13] LaMonte M.J., FitzGerald S.J., Church T.S., Barlow C.E., Radford N.B., Levine B.D., Pippin J.J., Gibbons L.W., Blair S.N., Nichaman M.Z. (2005). Coronary Artery Calcium Score and Coronary Heart Disease Events in a Large Cohort of Asymptomatic Men and Women. Am. J. Epidemiol..

[14] Heuvelmans M.A., Vonder M., Rook M., Groen H.J.M., De Bock G.H., Xie X., Ijzerman M.J., Vliegenthart R., Oudkerk M. (2019). Screening for Early Lung Cancer, Chronic Obstructive Pulmonary Disease, and Cardiovascular Disease (the Big-3) Using Low-Dose Chest Computed Tomography: Current Evidence and Technical Considerations. J. Thorac. Imaging.

[15] Ostrowski M., Marjański T., Dziedzic R., Jelitto-Górska M., Dziadziuszko K., Szurowska E., Dziadziuszko R., Rzyman W. (2019). Ten Years of Experience in Lung Cancer Screening in Gdańsk, Poland: A Comparative Study of the Evaluation and Surgical Treatment of 14 200 Participants of 2 Lung Cancer Screening Programmes. Interact. Cardiovasc. Thorac. Surg..

[16] Wood D.E. (2015). National Comprehensive Cancer Network (NCCN) Clinical Practice Guidelines for Lung Cancer Screening. Thorac. Surg. Clin..

[17] Mancia G., Fagard R., Narkiewicz K., Redón J., Zanchetti A., Böhm M., Christiaens T., Cifkova R., De Backer G., Dominiczak A. (2013). 2013 ESH/ESC Guidelines for the Management of Arterial Hypertension: The Task Force for the Management of Arterial Hypertension of the European Society of Hypertension (ESH) and of the European Society of Cardiology (ESC). J. Hypertens..

[18] Zdrojewski T., Rutkowski M., Bandosz P., Gaciong Z., Jedrzejczyk T., Solnica B., Pencina M., Drygas W., Wojtyniak B., Grodzicki T. (2013). Prevalence and Control of Cardiovascular Risk Factors in Poland. Assumptions and Objectives of the NATPOL 2011 Survey. Kardiol. Pol..

[19] Chiles C., Duan F., Gladish G.W., Ravenel J.G., Baginski S.G., Snyder B.S., DeMello S., Desjardins S.S., Munden R.F. (2015). Association of Coronary Artery Calcification and Mortality in the National Lung Screening Trial: A Comparison of Three Scoring Methods. Radiology.

[20] Shemesh J., Henschke C.I., Shaham D., Yip R., Farooqi A.O., Cham M.D., McCauley D.I., Chen M., Smith J.P., Libby D.M. (2010). Ordinal Scoring of Coronary Artery Calcifications on Low-Dose CT Scans of the Chest Is Predictive of Death from Cardiovascular Disease. Radiology.

[21] Htwe Y., Cham M.D., Henschke C.I., Hecht H., Shemesh J., Liang M., Tang W., Jirapatnakul A., Yip R., Yankelevitz D.F. (2015). Coronary Artery Calcification on Low-Dose Computed Tomography: Comparison of Agatston and Ordinal Scores. Clin. Imaging.

[22] Jacobs P.C., Gondrie M.J.A., van der Graaf Y., de Koning H.J., Isgum I., van Ginneken B., Mali W.P.T.M. (2012). Coronary Artery Calcium Can Predict All-Cause Mortality and Cardiovascular Events on Low-Dose CT Screening for Lung Cancer. Am. J. Roentgenol..

[23] Rasmussen T., Køber L., Abdulla J., Pedersen J.H., Wille M.M.W., Dirksen A., Kofoed K.F. (2015). Coronary Artery Calcification Detected in Lung Cancer Screening Predicts Cardiovascular Death. Scand. Cardiovasc. J..

[24] Pletcher M.J., Tice J.A., Pignone M., McCulloch C., Callister T.Q., Browner W.S. (2004). What Does My Patient’s Coronary Artery Calcium Score Mean? Combining Information from the Coronary Artery Calcium Score with Information from Conventional Risk Factors to Estimate Coronary Heart Disease Risk. BMC Med..

[25] Ostrowski M., Marczyk M., Dziedzic R., Jelitto-Górska M., Marjański T., Pisiak S., Jędrzejczyk T., Polańska J., Zdrojewski T., Wojtyniak B. (2019). Lung Cancer Survival and Comorbidities in Lung Cancer Screening Participants of the Gdańsk Screening Cohort. Eur. J. Public Health.

[26] Ruparel M., Quaife S.L., Dickson J.L., Horst C., Burke S., Taylor M., Ahmed A., Shaw P., Soo M.-J., Nair A. (2019). Evaluation of Cardiovascular Risk in a Lung Cancer Screening Cohort. Thorax.

[27] McClelland R.L., Jorgensen N.W., Budoff M., Blaha M.J., Post W.S., Kronmal R.A., Bild D.E., Shea S., Liu K., Watson K.E. (2015). 10-Year Coronary Heart Disease Risk Prediction Using Coronary Artery Calcium and Traditional Risk Factors: Derivation in the MESA (Multi-Ethnic Study of Atherosclerosis) with Validation in the HNR (Heinz Nixdorf Recall) Study and the DHS (Dallas Heart Study). J. Am. Coll. Cardiol..

[28] Leigh A., McEvoy J.W., Garg P., Carr J.J., Sandfort V., Oelsner E.C., Budoff M., Herrington D., Yeboah J. (2019). Coronary Artery Calcium Scores and Atherosclerotic Cardiovascular Disease Risk Stratification in Smokers. JACC Cardiovasc. Imaging.

[29] Budoff M.J., Young R., Burke G., Jeffrey Carr J., Detrano R.C., Folsom A.R., Kronmal R., Lima J.A.C., Liu K.J., McClelland R.L. (2018). Ten-Year Association of Coronary Artery Calcium with Atherosclerotic Cardiovascular Disease (ASCVD) Events: The Multi-Ethnic Study of Atherosclerosis (MESA). Eur. Heart J..

[30] Piepoli M.F., Hoes A.W., Agewall S., Albus C., Brotons C., Catapano A.L., Cooney M.-T., Corrà U., Cosyns B., Deaton C. (2016). 2016 European Guidelines on Cardiovascular Disease Prevention in Clinical Practice: The Sixth Joint Task Force of the European Society of Cardiology and Other Societies on Cardiovascular Disease Prevention in Clinical Practice (constituted by representatives of 10 societies and by invited experts) Developed with the special contribution of the European Association for Cardiovascular Prevention & Rehabilitation (EACPR). Eur. Heart J..

[31] Ward E., Jemal A., Cokkinides V., Singh G.K., Cardinez C., Ghafoor A., Thun M. (2004). Cancer Disparities by Race/Ethnicity and Socioeconomic Status. CA Cancer J. Clin..

[32] Zieliński A. (2015). Inequalities in Health and Social Policy. Przegl. Epidemiol..

[33] Jha P., Peto R. (2014). Global Effects of Smoking, of Quitting, and of Taxing Tobacco. N. Engl. J. Med..

[34] Hurt R.D., Ebbert J.O., Muggli M.E., Lockhart N.J., Robertson C.R. (2009). Open Doorway to Truth: Legacy of the Minnesota Tobacco Trial. Mayo Clin. Proc..

[35] Pedersen J.H., Ashraf H., Dirksen A., Bach K., Hansen H., Toennesen P., Thorsen H., Brodersen J., Skov B.G., Døssing M. (2009). The Danish Randomized Lung Cancer CT Screening Trial—Overall Design and Results of the Prevalence Round. J. Thorac. Oncol. Off. Publ. Int. Assoc. Study Lung Cancer.

[36] Ru Zhao Y., Xie X., de Koning H.J., Mali W.P., Vliegenthart R., Oudkerk M. (2011). NELSON Lung Cancer Screening Study. Cancer Imaging Off. Publ. Int. Cancer Imaging Soc..

[37] Shao L., Yan A.T., Lebovic G., Wong H.H., Kirpalani A., Deva D.P. (2017). Prognostic Value of Visually Detected Coronary Artery Calcification on Unenhanced Non-Gated Thoracic Computed Tomography for Prediction of Non-Fatal Myocardial Infarction and All-Cause Mortality. J. Cardiovasc. Comput. Tomogr..

[38] Takx R.A.P., Išgum I., Willemink M.J., van der Graaf Y., de Koning H.J., Vliegenthart R., Oudkerk M., Leiner T., de Jong P.A. (2015). Quantification of Coronary Artery Calcium in Nongated CT to Predict Cardiovascular Events in Male Lung Cancer Screening Participants: Results of the NELSON Study. J. Cardiovasc. Comput. Tomogr..

[39] Greenland P., Blaha M.J., Budoff M.J., Erbel R., Watson K.E. (2018). Coronary Calcium Score and Cardiovascular Risk. J. Am. Coll. Cardiol..

[40] Yeboah J., McClelland R.L., Polonsky T.S., Burke G.L., Sibley C.T., O’Leary D., Carr J.J., Goff D.C., Greenland P., Herrington D.M. (2012). Comparison of Novel Risk Markers for Improvement in Cardiovascular Risk Assessment in Intermediate-Risk Individuals. JAMA.

[41] Budoff M.J., Nasir K., Kinney G.L., Hokanson J.E., Barr R.G., Steiner R., Nath H., Lopez-Garcia C., Black-Shinn J., Casaburi R. (2011). Coronary Artery and Thoracic Calcium on Noncontrast Thoracic CT Scans: Comparison of Ungated and Gated Examinations in Patients from the COPD Gene Cohort. J. Cardiovasc. Comput. Tomogr..

